# Vertical and Horizontal Vegetation Structure across Natural and Modified Habitat Types at Mount Kilimanjaro

**DOI:** 10.1371/journal.pone.0138822

**Published:** 2015-09-25

**Authors:** Gemma Rutten, Andreas Ensslin, Andreas Hemp, Markus Fischer

**Affiliations:** 1 Institute of Plant Sciences, University of Bern, Bern, Switzerland; 2 School of Agricultural, Forest and Food Sciences, Bern University of Applied Science, Zollikofen, Switzerland; 3 Oeschger Center for Climate Change Research, University of Bern, Bern, Switzerland; 4 Department of Plant Systematics, University of Bayreuth, Bayreuth, Germany; 5 Botanical Garden, University of Bern, Bern, Switzerland; 6 Senckenberg Gesellschaft für Naturforschung, Biodiversity and Climate Research Center (BiK-F), Frankfurt, Germany; University of California Davis, UNITED STATES

## Abstract

In most habitats, vegetation provides the main structure of the environment. This complexity can facilitate biodiversity and ecosystem services. Therefore, measures of vegetation structure can serve as indicators in ecosystem management. However, many structural measures are laborious and require expert knowledge. Here, we used consistent and convenient measures to assess vegetation structure over an exceptionally broad elevation gradient of 866–4550m above sea level at Mount Kilimanjaro, Tanzania. Additionally, we compared (human)-modified habitats, including maize fields, traditionally managed home gardens, grasslands, commercial coffee farms and logged and burned forests with natural habitats along this elevation gradient. We distinguished vertical and horizontal vegetation structure to account for habitat complexity and heterogeneity. Vertical vegetation structure (assessed as number, width and density of vegetation layers, maximum canopy height, leaf area index and vegetation cover) displayed a unimodal elevation pattern, peaking at intermediate elevations in montane forests, whereas horizontal structure (assessed as coefficient of variation of number, width and density of vegetation layers, maximum canopy height, leaf area index and vegetation cover) was lowest at intermediate altitudes. Overall, vertical structure was consistently lower in modified than in natural habitat types, whereas horizontal structure was inconsistently different in modified than in natural habitat types, depending on the specific structural measure and habitat type. Our study shows how vertical and horizontal vegetation structure can be assessed efficiently in various habitat types in tropical mountain regions, and we suggest to apply this as a tool for informing future biodiversity and ecosystem service studies.

## Introduction

In most habitats, vegetation provides the main structure of the environment. This complexity can facilitate biodiversity [ 1 ] and provide ecosystem services. Therefore, measures of vegetation structure are often used to measure restoration success [2] and conservation value. More complex, heterogeneous habitats provide more niches and microhabitats for a higher number of specialized species [3]. Plants provide much of this heterogeneity via their diverse and complex growth forms [4]. This variation in physical structure in turn shapes a range of micro-environments relevant to other organisms [5,6]. In savannas, for instance, tree crowns generate benign environments which facilitate the growth of other plants [7]. In tropical forests, tree-fall gaps promote heterogeneity in otherwise homogeneous closed canopy forests [8]. Thus, plants are important ecosystem engineers that generate habitat niches, such as light patches in canopies and understory, and more complex vegetation structure utilized by other organisms, for example birds [9], reptiles and small mammals [10]. In this context, distinguishing horizontal (variation of structure across a horizontal space) from vertical vegetation structure (variation in structure across a vertical space) is important, as some taxa, e.g. birds or bees may depend more on strong changes in horizontal structures (e.g. trees for nesting and open area for foraging) than other taxa [4].

The structure provided by plants supports delivering ecosystem services. For example, vegetation can delay precipitation run-off via canopy interception and thereby prevent flooding and provides resilience to erosion [11]. Also, complex forests have stronger mitigation effects on climate extremes than pastures do, through evaporative cooling of many additive leaf layers [12]. Furthermore, vegetation structural variables, such as the Leaf Area Index (LAI), can be used as proxies for productivity [13,14] and terrestrial carbon stocks [14]. Altogether, this suggests that measures of vegetation structure can serve as valuable indicators of biodiversity and ecosystem services, which can be used in ecosystem management and conservation. However, common methods to assess vegetation structure, such as forest inventories, are very laborious. Moreover, they are restricted to habitats with trees, making it difficult to include habitats without trees.

A great opportunity to compare various habitat types within relatively short distances is provided by elevation gradients found on tropical mountains. Elevation, or factors related to it such as temperature and precipitation, affect vegetation composition, species richness and structure [15]. Many studies over elevation gradients report unimodal, “hump-shaped” patterns of species richness [16–18], but monotonous increases and decreases have also been found [19,20]. However, most of these studies assessing vegetation structure over elevation were restricted to a limited number of, mainly forested, habitat types.

In this study, we assessed the vegetation structure in forests and more open habitat types, such as savannas and alpine vegetation, using consistent measures of vegetation structure. Mount Kilimanjaro in Tanzania, provides the perfect setting for this study as it harbors an extraordinary range of habitat types over a broad elevation gradient. Furthermore, Mount Kilimanjaro provides natural resources for the local and regional population, including water, timber and optimal agroforestry conditions [21]. Therefore, we aimed to quantify the vegetation structure of the 12 prominent natural and human-modified habitat types at Mt. Kilimanjaro, using mean values of vertical vegetation characteristics as well as their variation in space (horizontal measures). We compared vegetation structure for six natural habitat types including forests and open habitats, covering a broad, natural elevation gradient (3750 m). Moreover, we compared vegetation structure in natural and modified habitat types at four elevation zones: (1) savanna and maize fields, (2) lower montane forests, traditional home gardens, commercial coffee farms and grasslands, (3) montane *Ocotea* forest and selectively logged *Ocotea* forest and finally, (4) upper montane *Podocarpus* forest and burned *Podocarpus* forest. By comparing this broad range of habitat types we evaluate the advantages and disadvantages of the methods used and give recommendations for future vegetation structure studies.

## Methods

### Study area

**Fig 1:**
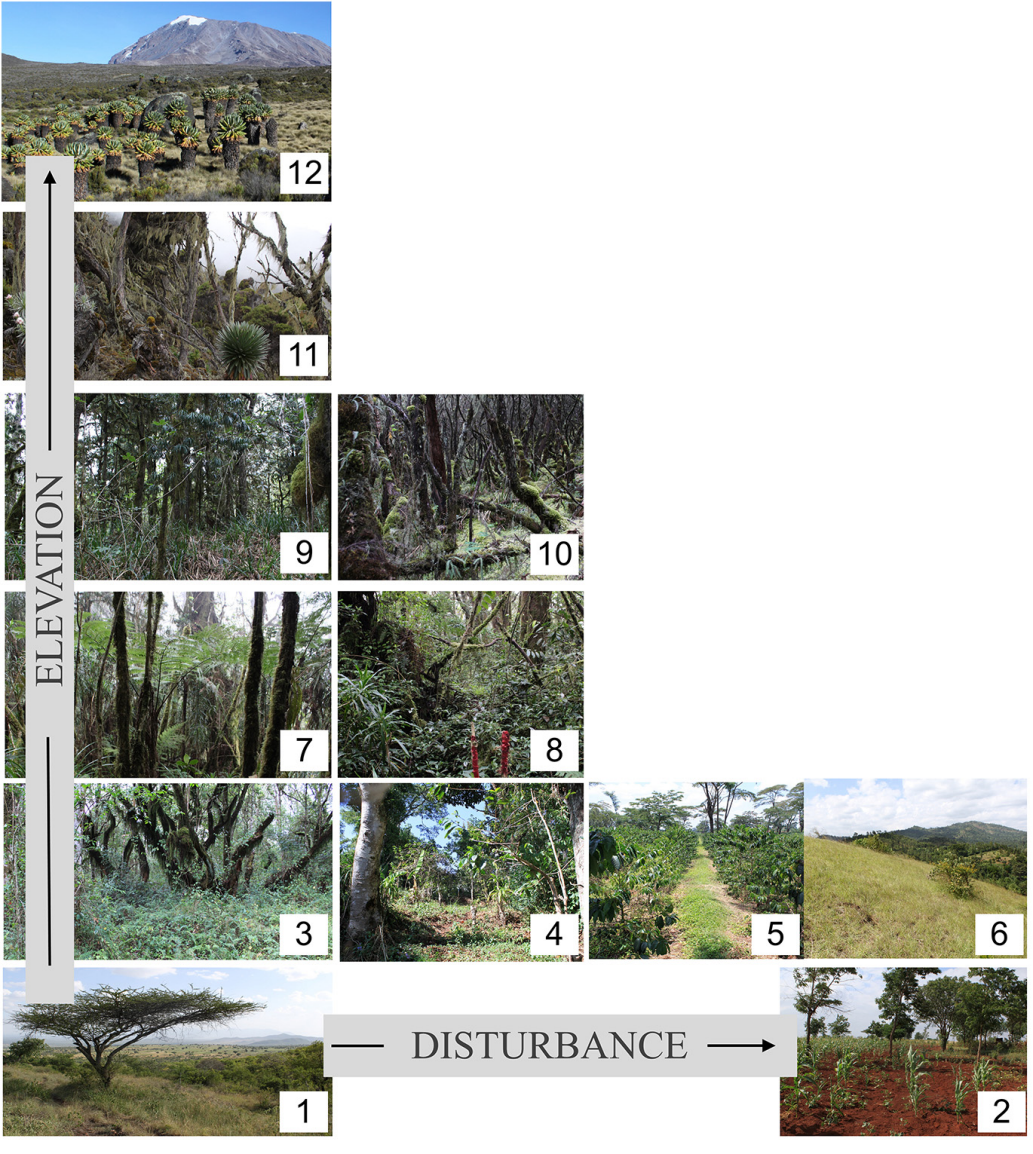
Mount Kilimanjaro’s main habitat types. Most of the **savanna** ^1^ zone (800–1100 m asl) is used for crop production (**maize** ^**2**^, beans and sunflowers). Similarly to the **lower montane** ^3^ forest zone (1100–2000 m asl) where many **home gardens** ^**4**^ (traditional agroforestry systems), commercial **coffee farms** ^5^ and **grasslands** ^**6**^ occur. In this zone, only few remnants of the natural forest vegetation are left. In the montane ***Ocotea*** ^***7***^ forest zone (2100 m to 2800 m asl), **selective logging** ^8^ occurred until 30–60yr ago. In the upper montane ***Podocarpus*** ^**9**^ forest zone (2800–3100 m asl), considerable areas were destroyed by fires several decades ago, the tree *Erica excelsa* (Ericacea) has colonized these previously **burned** ^10^ areas. In the subalpine, ***Erica*** ^***11***^ *trimera* zone (3500–4000 m asl), Africa’s highest forests can be found. Higher up we find the alpine ***Helichrysum*** ^***12***^ zone (4000–4500 m asl). In our study, each habitat type was represented by five sites which were distributed over an east-west gradient along the southern mountain slope and elevation gradient within each vegetation zone.

Mount Kilimanjaro, Tanzania, is located 300 km south of the equator and stretches from the savannas at 800 m to the snow-covered summit 5895 m above sea level (asl). For this relatively small area, it contains a globally unique range of climatic and vegetation zones [22]. We studied the southern and south-eastern slopes because they have a distinctly wetter climate than the northern and western slopes, resulting in a stronger vegetation zonation and a stronger human influence [22]. Thus our study area harbors a wide range of habitat types ranging from savannas over tropical montane forests to alpine vegetation [22]. The mean annual temperature linearly decreases from about 24°C in the savanna to 3°C in the alpine zone at 4300m and to minus 7°C at the Uhuru-peak at 5895m, with occasional frost events above about 2800m [22]. Annual precipitation shows a unimodal pattern ranging from 590 to 2500 mm/year with a peak at about 2200m, exceeding the precipitation of other African high mountains [22,23]. The relatively mild climate in the lower montane area facilitates traditional farming and more intensive coffee farming [21]. Together with the ongoing population growth, this resulted in a range of (human) modified habitat types in the lower and mid elevation zones [22,21], including traditional agriculture, coffee farms and selectively logged forest (Fig 1).

### Habitat types of Mt. Kilimanjaro

A dry and hot savanna zone surrounds the mountain between 800 m and 1100 m. Most of this area has been converted to crop production (maize, and also beans and sunflowers) and only few remnant fragments of the natural savanna, with *Acacia* (Mimosaceae), *Terminalia* (Combretaceae), *Grewia* (Malvaceae), and *Combretum* (Combretaceae) trees, remain [22]. In this zone, we studied natural savannas and maize fields.

The lower montane zone lies between 1100 m and 2000 m.The lower regions of this zone harbor the highest human population density at the mountain (about 500 people/km^2^ in [22,24]. Most of this area was converted into the so-called “Chagga home gardens”, a traditional agroforestry system where a range of crops such as bananas, coffee and beans are grown while some original forest trees are retained for shading [24]. In addition, in this zone, former forest patches were turned into grasslands to feed livestock. Furthermore, commercial coffee farms occur in this zone. Today, remnants of the natural forest vegetation, which is dominated by the genera *Newtonia* (Fabaceae), *Strombosia* (Olacaceae), *Entandrophragma* (Meliaceae) and *Macaranga* (Euphorbiaceae), are almost exclusively restricted to steep valleys and gorges [22]. In this zone, we studied natural lower montane forests, traditional home gardens, commercial coffee farms and traditional cut grasslands.

In the montane *Ocotea* zone (2100 m to 2800 m), the forests are dominated by *Ocotea usambarensis* (Lauraceae) [22], which due to its high commercial value was the main target for earlier selective logging activities. Although there is still occasional illegal selective logging [25], most selective logging took place before 1984 [22]. In this zone, we studied natural *Ocotea* forests and the modified, selectively logged forests.

In the upper montane forest zone (2800 m to 3100 m), considerable areas of the natural *Podocarpus latifolius* (Podocarpaceae) forests have been burned during the last three decades. Post burning, the tree *Erica excelsa* (Ericacea) has colonized from higher elevation and now dominates these previously burned areas [26]. In this zone, we studied both natural *Podocarpus* forests and burned areas. In the subalpine zone we studied remaining patches of natural *Erica trimera* (Ericacea) forest, which are the highest elevation forests in Africa, reaching up to 4000 m. Finally, in the alpine zone, we studied the *Helichrysum* heathland, characterized by *Helichrysum* cushion plants and tussock grasses, which extends up to over 4500 m [21]. A more detailed description of the vegetation within these habitat types is provided in [21,22,24,26].

### Site selection

Between 2010 and 2011, we established 60 20 x 50 m sites (S1 Fig and S1 Table), five sites in each of the 12 most prominent natural and modified habitat types (see above; Fig 1). The five sites per habitat type were distributed over an east-west gradient along the southern slope and elevation gradient within each vegetation zone (S1 Fig and S1 Table). The location of the sites was chosen according to its representativeness for the respective habitat type, and according to accessibility and security (A. Hemp, personal expertise). The distances between the sites ranged from 0.3 km to 54 km (S1 Fig and S1 Table).

### Vertical and horizontal vegetation structure

To characterize vertical vegetation structure, we measured structural variables at 9 points in each site and calculated the mean for each variable per site. To get a realistic estimate of the structure in the whole 20 x 50 m area we distributed the points over the site using the same sampling scheme in each site (S2 Fig). At each point at each site, the first author measured the number of vertical layers, effective layer width, vegetation density, maximum vegetation height, and maximum vegetation cover and the second author measured the LAI (Table 1). We measured width and height of each leaf layer by pointing to the beginning and end of each leaf layer at every measure point using a laser rangefinder (TruPulse 200/200, Centennial, USA). We calculated vegetation density by dividing total layer width by maximum vegetation height. We estimated percentage vegetation cover per square meter for each layer. We measured the LAI at ground level using a plant canopy analyzer in combination with a remote ‘above canopy’ sensor (LAI 2200, LI-COR Bioscience USA, 2011). The ‘above canopy’ reading was measured at ground level in an open area or forest gap as close to the site as possible and LAI values were calculated using the program FV-2200 (LI-COR Bioscience USA, 2011).

**Table 1:** Vertical and horizontal vegetation structure variables with description and method. The correlation matrix presents Spearman’s rank correlations across the 30 sites in natural habitats at Mount Kilimanjaro. A correlation coefficient of > 0.7 of highly correlated variables is highlighted in bold. Sub-column names (1/6) in vertical and horizontal vegetation structure represent the variables in the respective rows of the first column, where they are named. This order is also consistent with other tables and figures.

Variable			Description	Method	vertical structure						horizontal structure					
**Vertical structure**					1	2	3	4	5	6	1	2	3	4	5	6
	Number of layers	1	count of layers	visual	1.0	**0.8**	**0.7**	**0.8**	**0.7**	**0.8**	0.1	0.4	0.1	**0.8**	**0.8**	**0.8**
	Effective layer width	2	sum start and end layers	rangefinder	**0.8**	1.0	**0.7**	**1.0**	**0.8**	**0.8**	0.2	0.4	0.0	**0.8**	**0.8**	**0.7**
	Leaf density (width/height)	3	vertical leaf column	calculation	**0.7**	**0.7**	1.0	**0.8**	**0.7**	**0.7**	0.2	0.5	0.2	**0.7**	**0.8**	**0.7**
	Maximum vegetation height	4	Highest leaf	rangefinder	**0.8**	**1.0**	**0.8**	1.0	**0.7**	**0.7**	0.2	0.4	0.1	**0.7**	**0.8**	**0.7**
	Leaf Area Index (LAI)	5	Leaf area index	Licor	**0.7**	**0.8**	**0.7**	**0.7**	1.0	**0.8**	0.2	0.4	0.1	**0.8**	**0.9**	**0.8**
	Maximum vegetation cover	6	vegetation cover on m2	visual	**0.8**	**0.8**	**0.7**	**0.7**	**0.8**	1.0	0.1	0.5	0.1	**0.8**	**0.9**	**0.9**
**Horizontal structure**																
	CV number of layers	1	variability in layering	calculation	0.1	0.2	0.2	0.2	0.2	0.1	1.0	0.5	**0.7**	0.5	0.1	0.2
	CV layer width	2	variability layer width	calculation	0.4	0.4	0.5	0.4	0.4	0.5	0.5	1.0	0.4	**0.8**	0.4	0.6
	CV vegetation density	3	variability layer density	calculation	0.1	0.0	0.2	0.1	0.1	0.1	**0.7**	0.4	1.0	0.3	0.2	0.2
	CV vegetation height	4	variability effective layer height	calculation	**0.8**	**0.8**	**0.7**	**0.7**	**0.8**	**0.8**	0.5	**0.8**	0.3	1.0	**0.8**	**0.8**
	CV LAI	5	variability leaf area index	calculation	**0.8**	**0.8**	**0.8**	**0.8**	**0.9**	**0.9**	0.1	0.4	0.2	**0.8**	1.0	**0.8**
	CV vegetation cover	6	variability vegetation cover	calculation	**0.8**	**0.7**	**0.7**	**0.7**	**0.8**	**0.9**	0.2	0.6	0.2	**0.8**	**0.8**	1.0

To assess horizontal structure or spatial heterogeneity we used standard deviation (SD) as an absolute measure of variability and the coefficient of variation (CV) as a relative measure independent of the mean. These variables were calculated for each measure separately.

### Analysis

To test whether vertical and horizontal vegetation structure measures differ across natural habitats over elevation, we compared the 30 sites of 6 natural habitat types over the elevation gradient using regression analyses. We sought the best fitting linear model over elevation, using *R* ^*2*^ as the measure of fit and allowing for a quadratic term. Quadratic models were preferred over linear ones if they significantly improved the model fit in a likelihood ratio test.

To test whether vegetation structure was affected by habitat modification, we compared natural and modified habitat types in the (1) savanna zone, (2) lower montane zone, (3) *Ocotea* zone, and (4) *Podocarpus* zone, with separate ANOVAs for each zone. To test for differences between the four habitats in the lower montane zone, we performed Post-Hoc tests (Tukey's ‘Honest Significant Difference’ method). Prior to the analysis we tested for normality of all variables. All data were analyzed with *R*, Version 3.0.3 [27].

## Results

### Relation between elevation and structure

**Fig 2:**
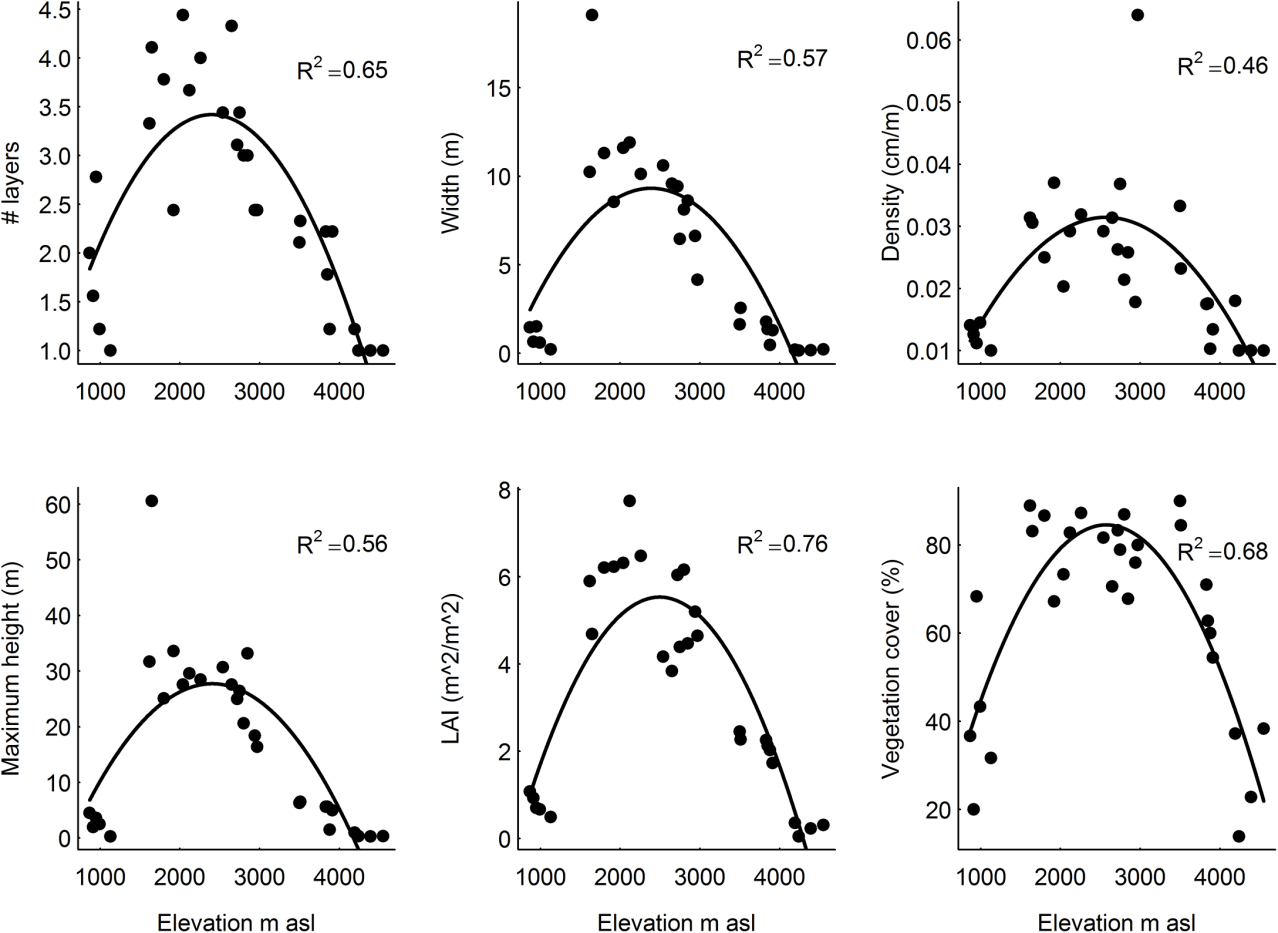
Relationship between elevation and vertical vegetation structure variables at Mt. Kilimanjaro, assessed as average of the structure variables among nine points in each of 30 sites. The fitted quadratic functions indicate significant relationships (P < 0.05 level).

**Fig 3:**
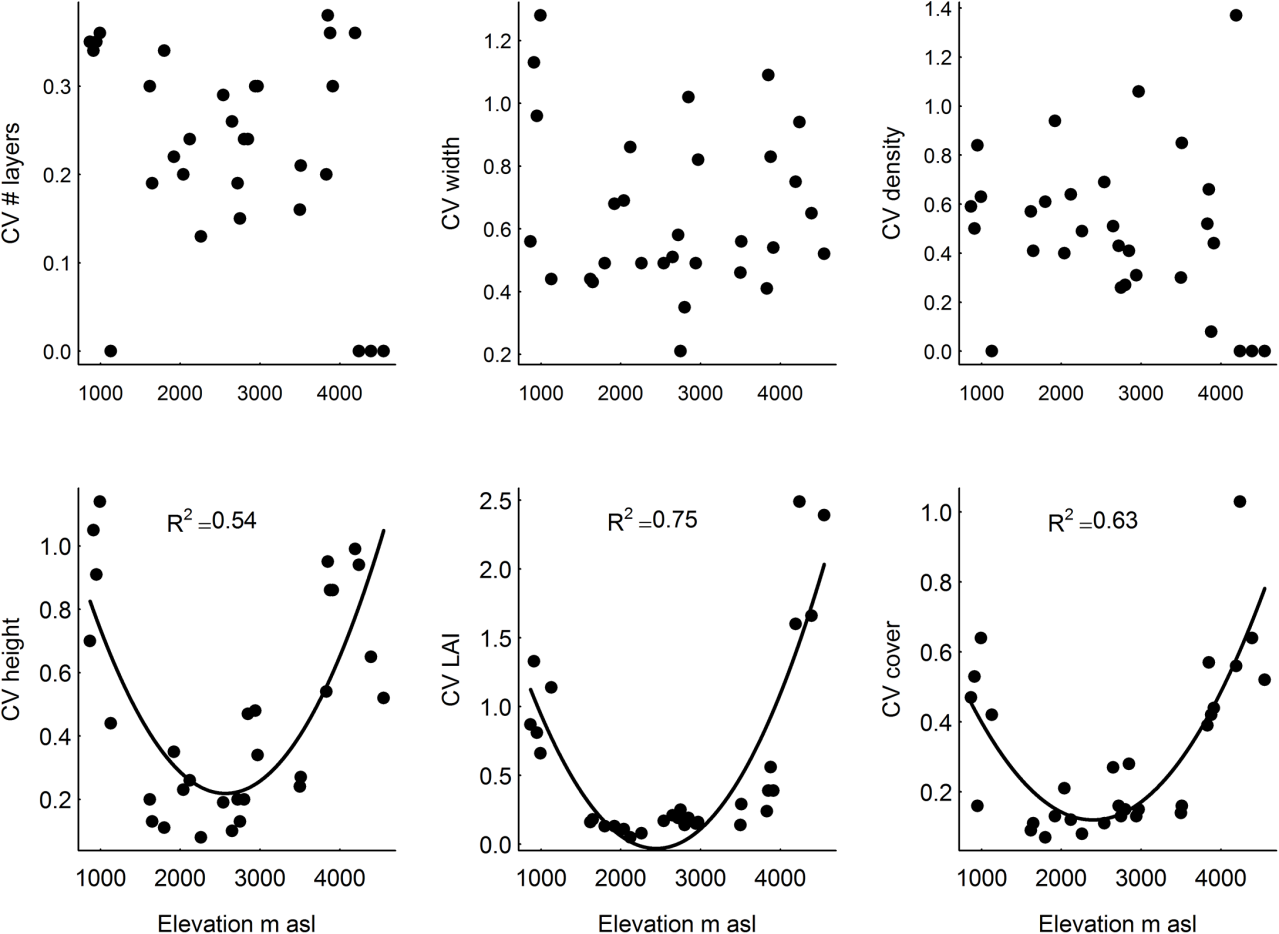
Relationship between elevation and horizontal vegetation structure variables at Mt. Kilimanjaro, assessed as the coefficient of variation (CV) of the structure variables among nine points in each of 30 sites. The fitted quadratic functions indicate significant relationships (P < 0.05 level).

All six vertical vegetation structure variables showed a unimodal pattern along the elevation gradient peaking at intermediate elevations (Table 2; Fig 2). Elevation explained between 45% (for vegetation density) and 75% (for LAI) of the variation in vertical vegetation structure. We assessed the six horizontal structure variables as absolute variation (SD) and relative variation independent of the mean (CV). As the absolute variation showed similar, though less pronounced, patterns as the means (S3 Fig), we chose to focus on the relative variation (CV) to assess the horizontal structure. Three of six horizontal vegetation structure variables showed an inverse hump-shaped pattern with lowest values at intermediate elevation (Table 2; Fig 3, CV of vegetation height, R^2^ = 0.54; CV of LAI R^2^ = 0.75, CV of vegetation cover R^2^ = 0.63). The remaining three variables of horizontal vegetation structure did not change systematically across elevation (Table 2; Fig 3, CV of number of layers, CV of layer width, CV of layer density).

**Table 2:** ANOVA results comparing vertical and horizontal vegetation structure variables among the a) six natural habitat types along the elevation gradient and for modified habitat types in each elevation zone of Mt. Kilimanjaro, b) savanna vs. maize fields, c) lower montane forests vs. home gardens vs. coffee farms vs. grassland, d) natural vs. previously selectively logged *Ocotea* forests, and e) natural vs. previously burned *Podocarpus* forests. Significant differences are shown in bold.

Variable	a) Elevation				b) Savanna			c) Lower montane			d) Ocotea			e) Podocarpus		
**Vertical structure**	_Df_	F value	*P*value	R^2^	_Df_	F value	*P*value	_Df_	F value	*P*value	_Df_	F value	*P*value	_Df_	F value	*P*value
Number of layers	_5;24_	24.18	**<0.001**	0.65	_1;5_	1.07	0.349	_3;16_	29.13	**<0.001**	_1;8_	1.17	0.310	_1;8_	0.85	0.384
Effective layer width	_5;24_	30.35	**<0.001**	0.57	_1;5_	0.02	0.893	_3;16_	18.01	**<0.001**	_1;8_	10.51	**0.012**	_1;8_	6.46	**0.035**
Leaf density (width/height)	_5;24_	5.25	**0.002**	0.45	_1;5_	1.53	0.271	_3;16_	7.67	**0.002**	_1;8_	0.41	0.539	_1;8_	0.2	0.664
Maximum height	_5;24_	26.1	**<0.001**	0.55	_1;5_	1.57	0.266	_3;16_	11.75	**<0.001**	_1;8_	4.19	0.075	_1;8_	11.46	**0.010**
Leaf Area Index (LAI)	_5;24_	37.57	**<0.001**	0.75	_1;4_	0.14	0.725	_3;16_	78.63	**<0.001**	_1;8_	1.77	0.221	_1;8_	6.41	**0.035**
Maximum cover	_5;24_	13.17	**<0.001**	0.67	_1;5_	1.46	0.281	_3;16_	4.55	**0.017**	_1;8_	0.09	0.774	_1;8_	2.26	0.171
**Horizontal structure**																
CV number of layers	_5;24_	0.83	0.539	0.05	_1;5_	0.18	0.690	_3;16_	4.16	**0.023**	_1;8_	0.73	0.418	_1;8_	0.77	0.407
CV layer width	_5;24_	1.43	0.250	0.09	_1;5_	0.98	0.368	_3;16_	1.97	0.159	_1;8_	0.11	0.753	_1;8_	3.31	0.106
CV vegetation density	_5;24_	0.48	0.790	0.00	_1;5_	0.01	0.934	_3;16_	11.89	**<0.001**	_1;8_	2.51	0.152	_1;8_	0.04	0.851
CV vegetation height	_5;24_	10.27	**<0.001**	**0.54**	_1;5_	0.37	0.572	_3;16_	10.2	**0.001**	_1;8_	0.08	0.786	_1;8_	8.89	**0.018**
CV LAI	_5;24_	18.37	**<0.001**	**0.75**	_1;4_	3.5	0.135	_3;16_	5.73	**0.007**	_1;8_	1.87	0.209	_1;8_	0	0.950
CV vegetation cover	_5;24_	9.24	**<0.001**	**0.63**	_1;5_	1.82	0.235	_3;16_	4.92	**0.013**	_1;8_	0.31	0.595	_1;8_	4.43	0.068

### Relation between habitat disturbance and structure

**Fig 4:**
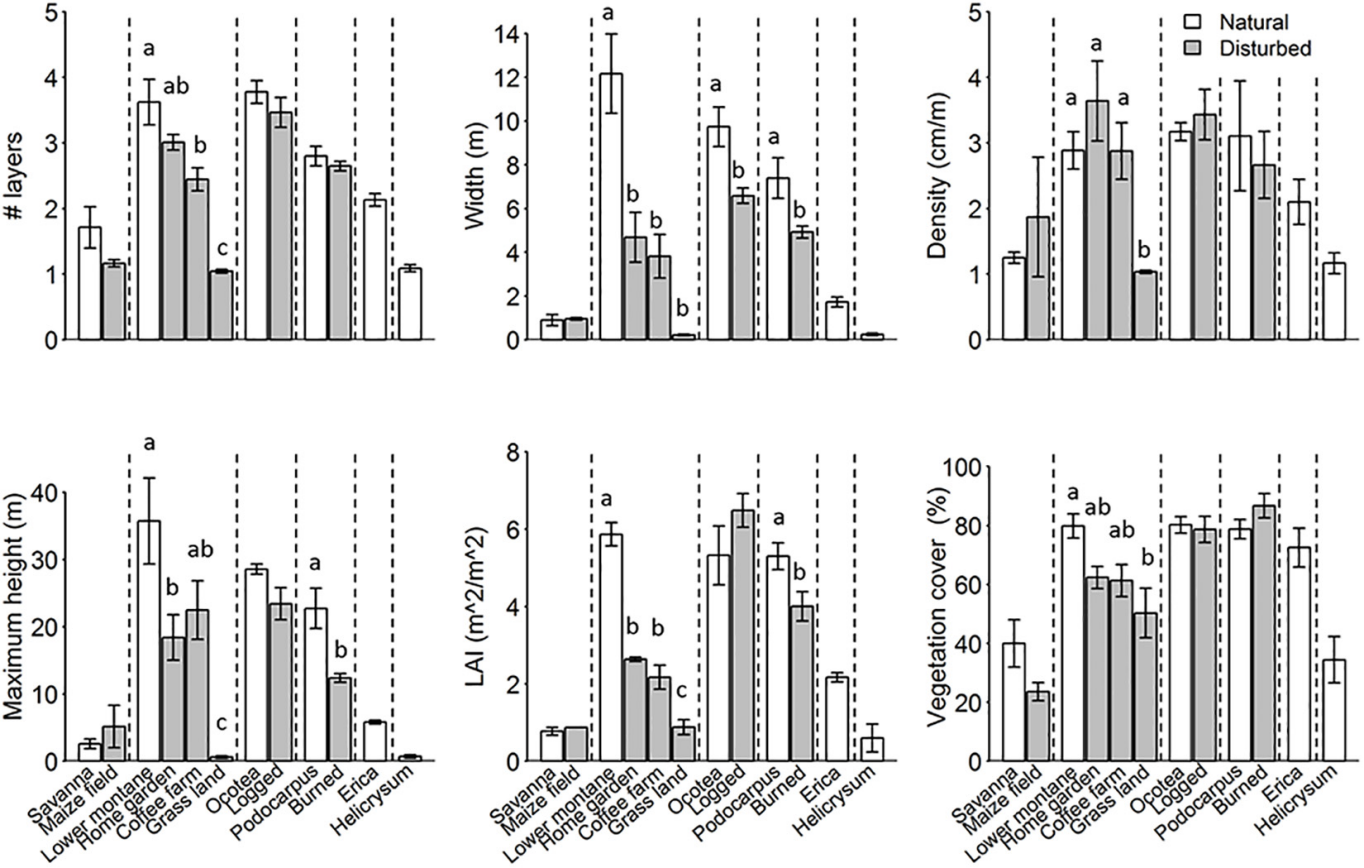
Vertical vegetation structure for each of 12 habitat types (each represented by five study sites) at Mt. Kilimanjaro. Bars indicate means ±SE for the six natural habitat types (white bars) and the six modified habitat types (grey bars). Dashed lines separate different elevation zones. Bars not sharing a character are significantly different from each other (P < 0.05 level).

**Fig 5:**
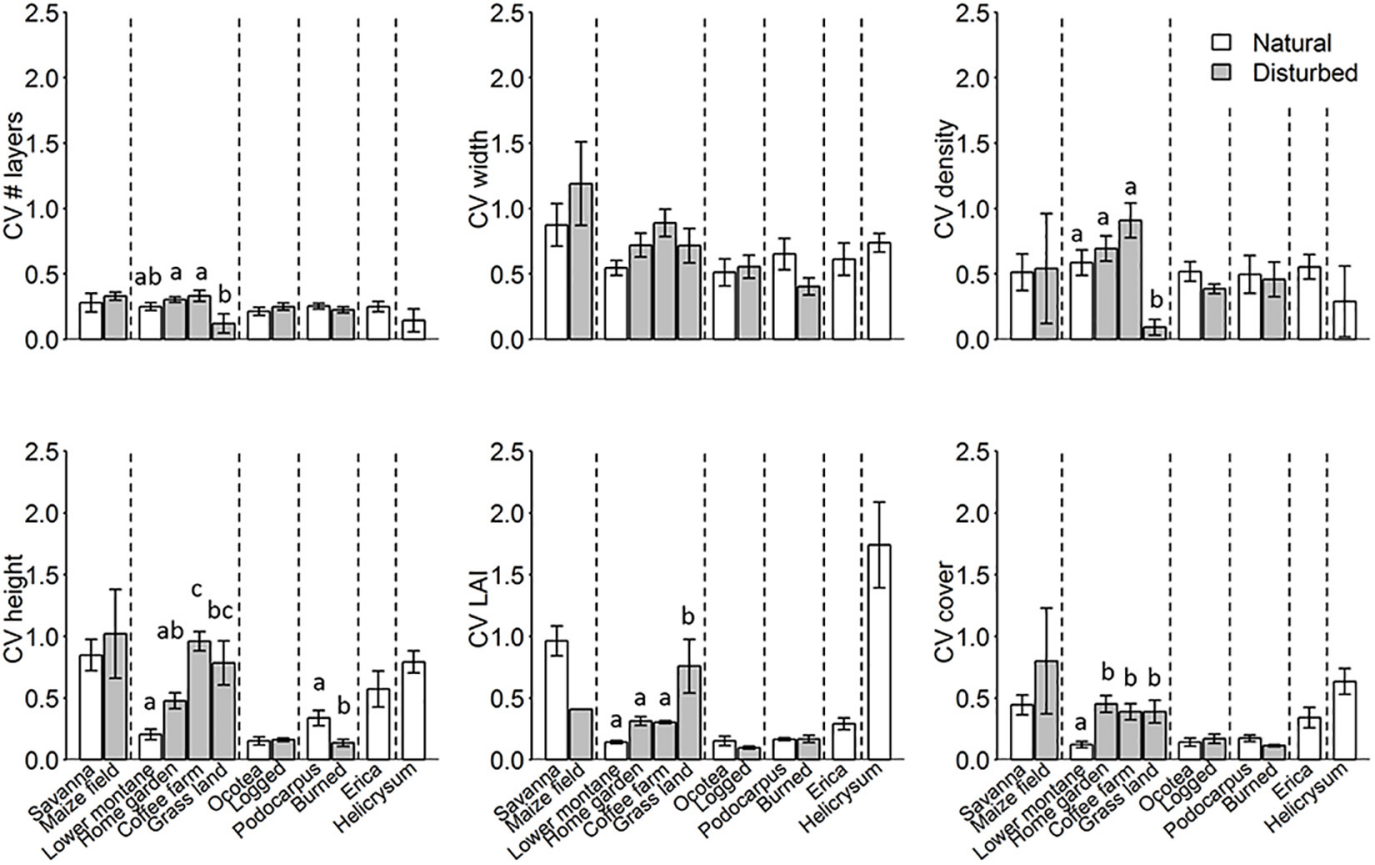
Horizontal vegetation structure (coefficients of variation (CV) of the structure variables per site) for each of 12 habitat types (each represented by five study sites) at Mt. Kilimanjaro. Bars indicate means ±SE for the six natural habitat types (white bars) and the six modified habitat types (grey bars). Dashed lines separate different elevation zones. Bars not sharing a character are significantly different from each other (P < 0.05 level).

Overall, the vertical vegetation structure measures were either lower or not significantly different in modified habitat types than in natural ones (Fig 4, Table 2). In the savanna zone, we could not detect significant differences between savanna and maize fields in the measured variables, neither in the vertical and horizontal structure, nor in the LAI (Table 2B; Figs 4 and 5).

In the lower montane zone, vertical structure measures were lower in human modified habitat types than in the natural forest (Table 2C; Fig 4). All vertical structure measures were consistently lower in grassland than in natural forest. Moreover, layer width and LAI were lower in home gardens and coffee farms than in natural forest. The number of layers was similar in home gardens and forests, while it was lower in coffee farms than in the forests. Whereas maximum height was similar in coffee farms and forests, it was lower in the home gardens than in the forest (Table 2). Thus, different anthropogenic modifications had different effects on vertical vegetation structure, where especially the grasslands showed lower vertical vegetation structure compared with the natural forest.

In contrast to vertical structure, all but one of the horizontal structure variables (Table 2C; Fig 5; CV of number of layers, CV of layers width, CV of canopy height; CV of LAI and CV of vegetation cover) were higher or similar in modified habitats than in the natural lower montane forest. The only exception was the CV of vegetation density, which was lower in grasslands than in the three other habitat types (forests, home gardens and coffee farms).

In the montane *Ocotea* zone, most vertical vegetation structure measures were not significantly different between the natural and selectively logged forests (Table 2D; Fig 4; number of layers, density, height, LAI, vegetation cover,). Only the layer width was lower in selectively logged forests than in natural forests. Also the horizontal structure variables showed no differences between natural and selectively logged forests (Table 2D; Fig 5). This indicates that selectively logged forests are as heterogeneous at small scales as natural *Ocotea* forests are.

In the upper montane *Podocarpus* zone, three of six vertical vegetation structure variables were lower in burned forests than in natural forests (Table 2E; Fig 4, layer width, vegetation height and LAI). However, burned and natural *Podocarpus* forests did not show differences in most horizontal structure variables (Table 2E; Fig 5; CV of number of layers, CV of layers width, CV of canopy density; CV of LAI and CV of vegetation cover). The only exception was the CV of vegetation height, which was lower in burned forests than in natural forests.

## Discussion

### Unimodal patterns of natural vegetation structure over elevation

Many of previous studies along elevation gradients showed unimodal patterns of plant species richness over elevation [16–18,28]. Most of the studies measuring both vegetation structure and plant species diversity, showed a hump-shape pattern over elevation for both. Similar patterns were found for plant species richness at Mount Kilimanjaro[22], as well as for biomass and carbon stocks [29]. This suggests that our vertical structure measures over elevation (Fig 2) could be used as a proxy for plant species richness, biomass and carbon stocks, at least at a broad scale across habitat types. Additionally, our LAI results suggest a peak in productivity at intermediate elevations. As habitats with a more complex vegetation structure catch more water than habitats with less complex vegetation [11], we might also expect an important role of Kilimanjaro's forests in regional water cycles, in line with the previously proposed important role of Kilimanjaro as a water tower [30].

Contrary to the consistent elevation pattern among vertical structure variables in natural habitats (Fig 4), only half of the horizontal structure variables showed a unimodal pattern over elevation for natural habitats (Fig 5). These three variables (CV of height, CV of LAI and CV of vegetation cover) showed an inverse hump shape which was lowest at intermediate elevation. Thus, among natural habitats horizontal structure components were more pronounced in more open habitats, in line with the idea of increased spatial heterogeneity in habitats with rare structural features, such as sparse savanna trees [31].The habitat types with a variable vertical vegetation structure and pronounced horizontal vegetation structure, in the lowest and highest zones of the mountain might be especially important for animal taxa that have various habitat requirements for various behavior. For example, savanna grazers need grass to forage as well as an occasional shading tree to keep from the heat [32] and insectivorous bats and birds might use open areas to forage, but they need complex structures (e.g. trees and tree holes) to roost and nest [7]. Furthermore, many insects (bees and wasps) and reptiles (snakes and lizards) need patches for shelter and patches to warm up in the sun before they become active. Therefore, vegetation patchiness in both vertical and horizontal space might be important [33]. Thus, considering both vertical and horizontal vegetation structure is needed to account comprehensively for the habitat requirements of different organisms. Therefore, the savanna and alpine habitats, harboring sites of different vertical vegetation structure and of pronounced horizontal vegetation structure, might each harbor diverse groups of organisms with more horizontal patchy habitat requirements, suggesting that they might also have considerable conservation value.

### Habitat modification affects vegetation structure

In the savanna zone we found no differences between natural savanna and maize fields in any of the vertical or horizontal vegetation structure variables. The maize fields in our study are mostly owned by small-scale farmers, who retain occasional trees and shrubs on their lands, which might serve similar purposes as the scattered trees in the savannas, as our horizontal structure data suggest (only two of five maize sites were completely treeless). However, Ensslin and colleagues[29] found a decrease in biomass of trees and shrubs and an increase of herbaceous biomass in the same maize fields and savannas, suggesting that differences may be more subtle and depend on the method and scale of measurement.

The pronounced horizontal structure in the maize fields and natural savannas suggest that these habitat types could provide a diverse range of niches in the savanna zone, especially considering taxa that depend on a complex horizontal structure, such as birds needing distinct structures for nesting and for foraging [7].

In the lower montane zone, we found the strongest differences between natural and modified habitat types; nearly all vertical structure variables were lower in the modified habitat types (especially in grasslands) than in the natural forests. However, some vertical measures (vegetation density and vegetation cover) and most of the horizontal vegetation structure measures did not differ between the habitat types with trees, i.e. between forests, home gardens and coffee farms. Nevertheless, earlier findings that home gardens have a more complex structure than forests, whereas coffee farms are more homogenous [24], were clearly confirmed by our method. Agroforestry systems, such as the multi-layered home gardens in Java, have a similar complex vertical vegetation structure as secondary forests do [34], and shaded coffee plantations in Mexico are also valuable for conservation [35]. Clearly, the structural complexity of agroforestry systems strongly depends not only on the type, but also on the intensity of management, as found in Cameroon [36], and Indonesia [37]. Our study confirms complex vegetation structures in agroforestry systems at Mt. Kilimanjaro, suggesting that these systems might harbor considerable biodiversity and associated ecosystem services, e.g. carbon stocks [29]. This idea is also illustrated by flying fox diversity, which was similar in forests, home gardens and coffee plantations [38], in turn indicating similar seed dispersal services. This suggests that the management of these habitat types should not be intensified.

In the *Ocotea* zone, the similar vegetation structure in natural and previously selectively logged forests indicates that the structure has recovered to a large extent. In a parallel study, using the same sites, the natural and previously selectively logged forests were also similar in biomass and carbon stock [29], showing that previously selectively logged forests are also important for carbon storage. Moreover, based on their structural complexity, former selectively logged forests might also have a considerable value for conservation [39]. Generally, our results suggest forest structure recovery 30–60 years after selective logging, in line with recovery periods of 24 years reported for Central Africa [40] and of 14 years in Cameroon [41]. However, effects of past selective logging differed strongly between sites, some sites being completely depleted from the formerly dominant target species *Ocotea usambarensis* [42]. Moreover, layer width was still narrower in the selectively logged forests suggesting increased light availability possibly enhancing recruitment [42,43]. Further studies should address such a possible recruitment boost resulting from different selective logging intensities and histories, as effective recruitment matters largely for the resilience of a forest to disturbance.

The differences between natural and formerly burned forests in the *Podocarpus* zone were more pronounced than in the *Ocotea* zone and apparent in lower values of three measures of vertical structure. Formerly burned areas had lower canopy height, leaf width and higher below-canopy light availability. This indicates that canopies in burned *Podocarpus* forests remain more open than their natural counterparts, even after 30 years. Also one measure of horizontal structure (CV of canopy height) was lower in the burned than in the natural forests. This corresponds well with earlier findings of a strong change in species composition in burned areas [24]. Along with the changes in plant composition, the decreases in structural diversity may result in a decrease in species richness of other trophic levels due to a reduction of niche diversity in the burned areas compared to natural *Podocarpus* forest.

### Methodical considerations

As recognized by many authors before, both vertical as well as horizontal vegetation structure are not well defined concepts. Consequently, they can be measured in many different ways [4,44,45]. We showed that no single variable for structure can represent all others. Still, the direction of disturbance and elevation effects on most measures was largely similar, though not always significantly so. Therefore, we recommend the method employed here as an appropriate alternative to more laborious techniques that require expert-knowledge, such as forest inventories.

Specifically, when very fast and efficient assessments of many habitat types at a very large scale are required, we would recommend to use number of leaf layers or vegetation cover across all layers. These visual measures are very straight-forward and fast [46] and efficient as they show strong correlations to all other measured variables (e.g. LAI-measurements or biomass calculations from forest inventories). Furthermore, they do not require expensive measuring devices and they do not depend on weather conditions; such as the devices for measuring the LAI and canopy height, which do not work when it rains. Additionally the LAI measures depend on cloud cover and on finding an appropriate reference area.

Vertical and small-scale horizontal vegetation structure affect the abundance and diversity of other organisms, such as seedlings [47], lichens [48], beetles [49], bees [50], moths [51], spiders [52], birds [9], bats [38,53] and reptiles and small mammals [10] as well as micro-environments [54]. So far the majority of studies relating animal diversity to habitat complexity focused on vertebrates, often in human-modified areas [4]. The applicability of our structural measures across a very broad range of different habitat types suggests that they can be used for studying the relationships between vegetation structure and the diversity of different plant, vertebrate, invertebrate, fungal and microbial taxa and of ecosystem processes over gradients of elevation and anthropogenic impact. Therefore, we suggest that both absolute values of vegetation characteristics (vertical measures) as well as their variation in space (horizontal measures) should be related to measures of biodiversity. At the same time this will elucidate their value as indicators of the conservation value and possibly the provisioning of ecosystem services.

## Conclusions

Our study quantifies vertical and horizontal vegetation structure for a very broad range of natural and modified habitats with relatively simple measures efficient in terms of labor and cost. We furthermore show that natural vegetation structure followed unimodal patterns over elevation on Mt. Kilimanjaro, with vertical structure highest at intermediate elevations and the horizontal structure lowest at intermediate elevation. Overall, modified habitat types had less pronounced vertical structure than natural types did, whereas horizontal structure responded inconsistently depending on specific habitat type, with a tendency to increased horizontal vegetation structure in more open habitats. We advocate the use of our simple and fast methods as they may assist assessments not only of vegetation structure across a broad range of habitat types, but also of indicators of biodiversity and ecosystem functioning.

## Supporting Information

S1 Fig Map of the study area. Indicates the sites at the southern and south-eastern slopes of Mt Kilimanjaro where the horizontal and vertical vegetation structure were studied. Coordinates are given in longitude and latitude using Geographical Coordinate System (WGS 84).
(DOCX)

S2 Fig Distribution of the nine sample points within each study site. (DOCX)

S3 Fig Relationship between elevation and horizontal structure variables assessed as the standard deviation (SD). The fitted quadratic functions indicate significant relationships (P < 0.05 level).
(DOCX)

S1 Table Habitat type, elevation, site mean and standard error of six vegetation structure variables measured in 9 sample points (S2 Fig) for each study sites. (DOCX)
