# Supplementary material for: Vertical and Horizontal Vegetation Structure across Natural and Modified Habitat Types at Mount Kilimanjaro
Source: PLoS One. 2015 Sep 25;10(9):e0138822. doi: 10.1371/journal.pone.0138822 (PMC4583428; doi:10.1371/journal.pone.0138822)

**S1. Fig. Map of the study area.** Indicates the sites at the southern and south-eastern slopes of Mt Kilimanjaro where the horizontal and vertical vegetation structure were studied. Coordinates are given in longitude and latitude using Geographical Coordinate System (WGS 84)


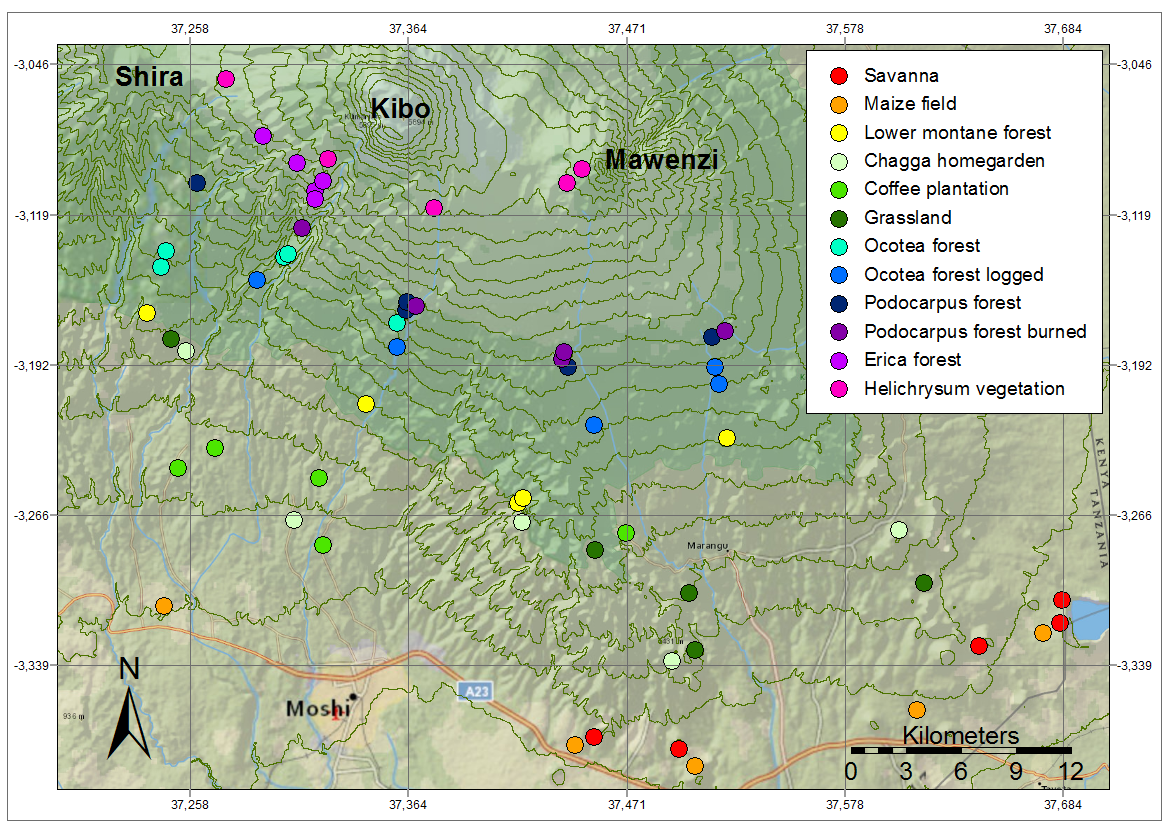

Supplement: S1 Fig — Indicates the sites at the southern and south-eastern slopes of Mt Kilimanjaro where the horizontal and vertical vegetation structure were studied. Coordinates are given in longitude and latitude using Geographical Coordinate System (WGS 84). (DOCX) [file pone.0138822.s001.docx]
