# Supplementary figures and images for: Vertical and Horizontal Vegetation Structure across Natural and Modified Habitat Types at Mount Kilimanjaro
Source: PLoS One. 2015 Sep 25;10(9):e0138822. doi: 10.1371/journal.pone.0138822 (PMC4583428; doi:10.1371/journal.pone.0138822)

**S2**. **Fig. Distribution of the nine sample points within each study site.**


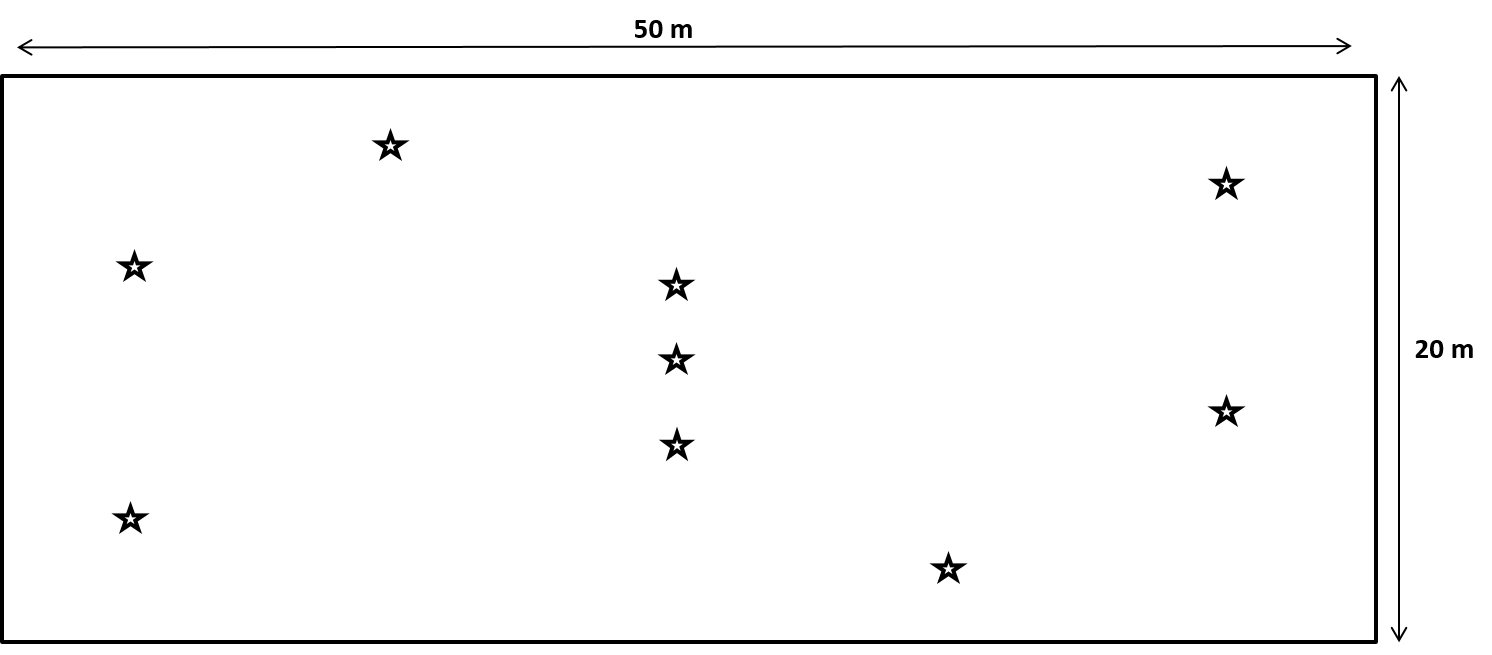

Supplement: S2 Fig — (DOCX) [file pone.0138822.s002.docx]
