# Supplementary material for: Vertical and Horizontal Vegetation Structure across Natural and Modified Habitat Types at Mount Kilimanjaro
Source: PLoS One. 2015 Sep 25;10(9):e0138822. doi: 10.1371/journal.pone.0138822 (PMC4583428; doi:10.1371/journal.pone.0138822)

**S3. Fig.** **Relationship between elevation and horizontal structure variables** **assessed as the standard deviation (SD).** The fitted quadratic functions indicate significant relationships (P < 0.05 level).


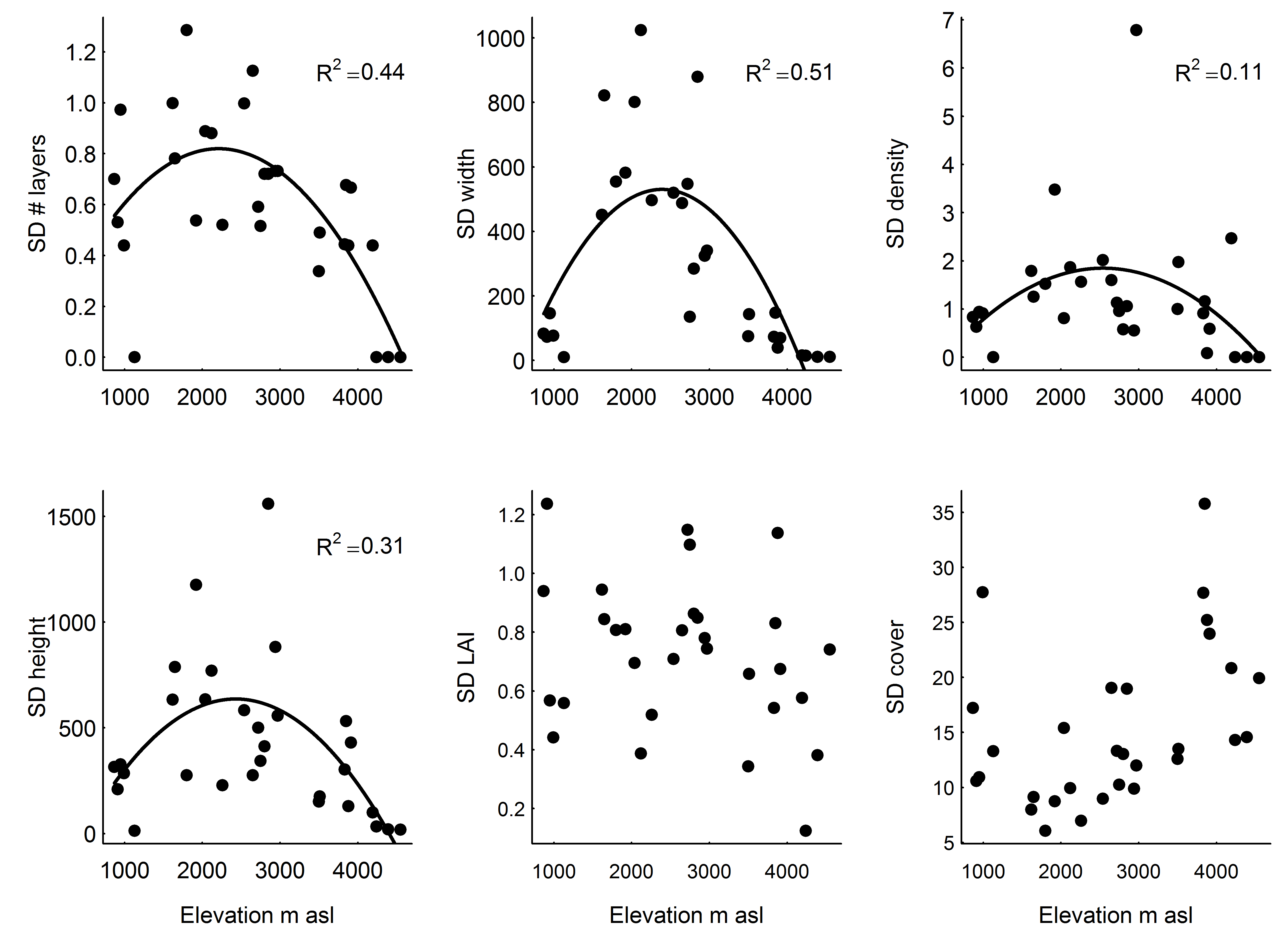

Supplement: S3 Fig — The fitted quadratic functions indicate significant relationships (P < 0.05 level). (DOCX) [file pone.0138822.s003.docx]
