# Supplementary material for: Vertical and Horizontal Vegetation Structure across Natural and Modified Habitat Types at Mount Kilimanjaro
Source: PLoS One. 2015 Sep 25;10(9):e0138822. doi: 10.1371/journal.pone.0138822 (PMC4583428; doi:10.1371/journal.pone.0138822)
Supplement: S1 Table — (DOCX) [file pone.0138822.s004.docx]

**S1 Table.** **Habitat type, elevation, site mean and standard error of six vegetation structure variables measured in 9 sample points (S2) for each study sites.**

| **Habitattype** | **Elevation (m.a.s.l.)** | **Number of layers** | | **Effective layer width** | | **Leaf Density** | | **Max. vegetation height** | | **Leaf Area Index** | | **Max. vegetation cover** | |
| --- | --- | --- | --- | --- | --- | --- | --- | --- | --- | --- | --- | --- | --- |
|  |  | mean(SE) | | mean(SE) | | mean(SE) | | mean(SE) | | mean(SE) | | mean(SE) | |
| SAV | 871 | 2 | (0.24) | 147.22 | (27.48) | 1.41 | (0.28) | 450 | (48.78) | 1.08 | (0.27) | 36.67 | (5.77) |
| SAV | 912 | 1.56 | (0.18) | 65 | (24.55) | 1.26 | (0.21) | 200 | (28.74) | 0.93 | (0.36) | 20 | (3.54) |
| SAV | 1130 | 1 | (0) | 22.22 | (3.24) | 1 | (0) | 30 | (3.24) | 0.49 | (0.16) | 31.67 | (4.41) |
| SAV | 993 | 1.22 | (0.15) | 59.89 | (25.63) | 1.45 | (0.3) | 250 | (33.06) | 0.67 | (0.12) | 43.33 | (9.28) |
| SAV | 950 | 2.78 | (0.32) | 152.33 | (48.71) | 1.12 | (0.31) | 360 | (49.67) | 0.7 | (0.16) | 68.33 | (3.54) |
| MAI | 1020 | NA |  | NA |  | NA |  | NA |  | NA |  | NA |  |
| MAI | 860 | NA |  | NA |  | NA |  | NA |  | NA |  | NA |  |
| MAI | 890 | NA |  | NA |  | NA |  | NA |  | NA |  | NA |  |
| MAI | 960 | 1.11 | (0.11) | 101.67 | (29.65) | 0.96 | (0.04) | 200 | (19.8) | 0.87 | (0.13) | 20.56 | (2.56) |
| MAI | 962 | 1.22 | (0.15) | 89.22 | (44.92) | 2.78 | (1.09) | 830 | (115.3) | NA |  | 26.67 | (10.9) |
| FLM | 1920 | 2.44 | (0.18) | 855.56 | (193.05) | 3.7 | (1.16) | 3360 | (220.29) | 6.23 | (0.21) | 67.22 | (2.9) |
| FLM | 1800 | 3.78 | (0.43) | 1130.56 | (185.47) | 2.5 | (0.5) | 2510 | (77.12) | 6.21 | (0.23) | 86.67 | (2.04) |
| FLM | 1620 | 3.33 | (0.33) | 1025 | (150.79) | 3.14 | (0.6) | 3170 | (175.67) | 5.9 | (0.26) | 88.89 | (2.61) |
| FLM | 1650 | 4.11 | (0.26) | 1910 | (272.23) | 3.06 | (0.42) | 6060 | (223.84) | 4.69 | (0.23) | 83.11 | (3.09) |
| FLM | 2040 | 4.44 | (0.29) | 1161.11 | (266.13) | 2.03 | (0.27) | 2760 | (145.97) | 6.32 | (0.19) | 73.33 | (5.2) |
| HOM | 1640 | 3.38 | (0.42) | 786.88 | (218.96) | 2.37 | (0.47) | 2710 | (311.57) | 2.57 | (0.18) | 76.25 | (5.41) |
| HOM | 1150 | 3.11 | (0.35) | 640.33 | (212.77) | 3.04 | (0.73) | 2160 | (243.41) | 2.72 | (0.24) | 62.78 | (9.79) |
| HOM | 1840 | 3 | (0.24) | 460 | (99.19) | 4.43 | (1.33) | 1570 | (96.36) | 2.79 | (0.17) | 61.11 | (9.71) |
| HOM | 1260 | 2.89 | (0.26) | 311.67 | (46.07) | 5.64 | (1.67) | 2060 | (230.72) | 2.65 | (0.29) | 57.78 | (9.79) |
| HOM | 1560 | 2.67 | (0.29) | 144.44 | (34.55) | 2.72 | (0.38) | 700 | (67.22) | 2.47 | (0.27) | 53.89 | (10.83) |
| COF | 1307 | 2.22 | (0.32) | 326.67 | (80.12) | 3.59 | (1.04) | 2460 | (368.44) | 1.38 | (0.12) | 60 | (9.61) |
| COF | 1360 | 2.67 | (0.29) | 219.44 | (59.42) | 2.47 | (0.57) | 1600 | (196.05) | 2.07 | (0.2) | 76.11 | (5.58) |
| COF | 1300 | 2.56 | (0.24) | 649.44 | (280.92) | 3.97 | (1.6) | 3260 | (434.28) | 1.65 | (0.14) | 51.67 | (9.46) |
| COF | 1120 | 1.89 | (0.26) | 138.33 | (34.6) | 1.51 | (0.28) | 920 | (113.37) | 2.77 | (0.24) | 47.78 | (7.03) |
| COF | 1660 | 2.89 | (0.2) | 577.22 | (163.4) | 2.85 | (1.15) | 3000 | (289.58) | 2.99 | (0.24) | 71.11 | (5.94) |
| GRA | 1660 | 1 | (0) | 20.56 | (4.03) | 1 | (0) | 40 | (4.03) | 0.94 | (0.17) | 73.89 | (3.51) |
| GRA | 1750 | 1.11 | (0.11) | 17.44 | (6.39) | 1.06 | (0.06) | 90 | (9.35) | 0.13 | (0.06) | 40 | (5.53) |
| GRA | 1480 | 1 | (0) | 11.11 | (1.62) | 1 | (0) | 20 | (1.62) | 1.07 | (0.18) | 39.44 | (6.43) |
| GRA | 1310 | 1 | (0) | 25 | (4.08) | 1 | (0) | 40 | (4.08) | 1.1 | (0.14) | 31.44 | (6.94) |
| GRA | 1300 | 1.11 | (0.11) | 35 | (11.24) | 1.11 | (0.11) | 120 | (14.58) | 1.17 | (0.15) | 66.67 | (5.53) |
| FOC | 2120 | 3.67 | (0.29) | 1190.56 | (340.12) | 2.92 | (0.62) | 2960 | (196.98) | 7.74 | (0.11) | 82.78 | (3.34) |
| FOC | 2260 | 4 | (0.17) | 1012.78 | (166.2) | 3.19 | (0.52) | 2850 | (69.44) | 6.48 | (0.14) | 87.22 | (2.22) |
| FOC | 2540 | 3.44 | (0.34) | 1060.56 | (171.8) | 2.92 | (0.67) | 3070 | (148.11) | 4.17 | (0.2) | 81.67 | (3) |
| FOC | 2650 | 4.33 | (0.37) | 957.22 | (162.63) | 3.14 | (0.53) | 2760 | (75.25) | 3.84 | (0.23) | 70.56 | (6.26) |
| FOC | 2750 | 3.44 | (0.18) | 645.56 | (45.46) | 3.68 | (0.32) | 2640 | (96) | 4.39 | (0.31) | 78.89 | (3.41) |
| FOD | 2220 | 3.78 | (0.22) | 772.22 | (207.61) | 3.44 | (0.41) | 2640 | (96.03) | 5.74 | (0.15) | 63.33 | (6.29) |
| FOD | 2470 | 3.44 | (0.38) | 608.89 | (133.96) | 4.73 | (0.74) | 3110 | (144.27) | 5.38 | (0.21) | 74.44 | (5.1) |
| FOD | 2270 | 2.78 | (0.28) | 576.67 | (111.9) | 3.67 | (0.54) | 2220 | (95.49) | 6.44 | (0.16) | 82.22 | (3.83) |
| FOD | 2560 | 4.11 | (0.31) | 631.11 | (67.85) | 2.74 | (0.24) | 1800 | (64.61) | 7.73 | (0.18) | 85.56 | (2.82) |
| FOD | 2370 | 3.22 | (0.22) | 702.78 | (95.58) | 2.58 | (0.35) | 1940 | (99.51) | 7.15 | (0.18) | 87.78 | (3.02) |
| FPO | 2850 | 3 | (0.24) | 862.22 | (292.28) | 2.58 | (0.35) | 3320 | (278.55) | 4.47 | (0.23) | 67.78 | (6.3) |
| FPO | 2940 | 2.44 | (0.24) | 662.22 | (108.85) | 1.78 | (0.18) | 1840 | (194.73) | 5.2 | (0.22) | 76 | (3.42) |
| FPO | 2970 | 2.44 | (0.24) | 415.56 | (114.15) | 6.4 | (2.26) | 1640 | (150.29) | 4.65 | (0.21) | 80 | (3.91) |
| FPO | 2720 | 3.11 | (0.2) | 943.33 | (183.23) | 2.63 | (0.38) | 2500 | (136.59) | 6.04 | (0.32) | 83.33 | (4.56) |
| FPO | 2800 | 3 | (0.24) | 812.22 | (94.89) | 2.14 | (0.19) | 2060 | (110.78) | 6.16 | (0.23) | 86.89 | (4.22) |
| FPD | 3060 | 2.67 | (0.17) | 568.56 | (70.16) | 2.17 | (0.21) | 1370 | (53.36) | 2.61 | (0.18) | 92.22 | (3.55) |
| FPD | 2990 | 2.89 | (0.31) | 436.67 | (92.98) | 4.68 | (1.48) | 1400 | (42.17) | 4.4 | (0.14) | 87.78 | (3.24) |
| FPD | 2880 | 2.56 | (0.18) | 550.56 | (43.64) | 2.07 | (0.15) | 1220 | (27) | 4.85 | (0.22) | 91.11 | (2.32) |
| FPD | 2820 | 2.44 | (0.18) | 440 | (54.31) | 2.44 | (0.43) | 1070 | (37.86) | 4.18 | (0.12) | 70.56 | (3.17) |
| FPD | 2770 | 2.67 | (0.17) | 465.56 | (62.25) | 1.96 | (0.2) | 1130 | (66.31) | 3.99 | (0.28) | 92 | (3.95) |
| FER | 3910 | 2.22 | (0.22) | 129.44 | (23.27) | 1.34 | (0.2) | 500 | (55.8) | 1.73 | (0.19) | 54.44 | (7.93) |
| FER | 3850 | 1.78 | (0.22) | 135.89 | (49.52) | 1.76 | (0.38) | 560 | (80.34) | 2.13 | (0.24) | 62.78 | (11.91) |
| FER | 3510 | 2.33 | (0.17) | 255.56 | (47.46) | 2.32 | (0.66) | 650 | (37.75) | 2.27 | (0.19) | 84.44 | (4.44) |
| FER | 3830 | 2.22 | (0.15) | 178.78 | (24.31) | 1.75 | (0.31) | 560 | (56.64) | 2.26 | (0.15) | 71 | (9.19) |
| FER | 3500 | 2.11 | (0.11) | 162.67 | (24.92) | 3.33 | (0.34) | 630 | (38.55) | 2.45 | (0.1) | 90 | (4.08) |
| HEL | 3880 | 1.22 | (0.15) | 47.22 | (13.07) | 1.03 | (0.03) | 150 | (14.41) | 2.03 | (0.28) | 60 | (8.37) |
| HEL | 4190 | 1.22 | (0.15) | 20.78 | (5.18) | 1.8 | (0.82) | 100 | (9.7) | 0.36 | (0.16) | 37.22 | (6.98) |
| HEL | 4240 | 1 | (0) | 15 | (4.71) | 1 | (0) | 35 | (4.71) | 0.05 | (0.04) | 13.89 | (4.77) |
| HEL | 4390 | 1 | (0) | 16.67 | (3.63) | 1 | (0) | 30 | (3.63) | 0.23 | (0.1) | 22.78 | (4.87) |
| HEL | 4550 | 1 | (0) | 21.67 | (3.73) | 1 | (0) | 35 | (3.73) | 0.31 | (0.21) | 38.33 | (6.67) |
